# Evidence for avoidance tendencies linked to anxiety about specific types of thinking

**DOI:** 10.1038/s41598-023-29834-z

**Published:** 2023-02-25

**Authors:** Richard J. Daker, Michael S. Slipenkyj, Adam E. Green, Ian M. Lyons

**Affiliations:** Department of Psychology, Georgetown University, Washington, D.C., USA

**Keywords:** Psychology, Human behaviour

## Abstract

Anxieties that are specific to a particular kind of thinking have been demonstrated for a variety of cognitive domains. One hypothesized consequence of these anxieties is reduced interest in pursuing activities and, consequentially, careers that involve the type of thinking in question in an effort to avoid engaging in that type of thinking. There is little research addressing this avoidance hypothesis, possibly because it is difficult to categorize pursuits as objectively “creative” or “spatial”. Here, we measured the perceptions that participants, themselves, hold about how much pursuits (careers and activities) involve different types of thinking. We developed a novel framework for calculating “affinity coefficients”, within-person associations between perceived cognitive involvement and interest across several pursuits. Having a negative creative affinity coefficient, for instance, means being less interested in pursuits the more they are perceived as involving creative thinking. Results across three separate cognitive domains (creativity, mathematics, and spatial reasoning) reliably showed that higher anxiety in a domain uniquely predicted a lower affinity coefficient in that domain, providing consistent evidence of avoidance tendencies linked to cognition-specific anxieties. These findings suggest that feeling anxious about particular types of thinking may play a significant role in shaping our interests, both big and small.

## Introduction

Feeling anxious about specific types of thinking (e.g., creative thinking, math, spatial reasoning, etc.) is often related to important real-world outcomes^1–4^. One of the most-studied consequences of what we refer to as “cognition-specific anxieties”, or a tendency to feel anxious when faced with situations that involve a specific type of thinking (e.g., mathematical thinking), is poor cognitive performance on tasks that involve the type of thinking in question^3–8^. However, while cognitive underperformance has gotten the bulk of the research attention in these literatures, another important predicted consequence of cognition-specific anxieties is avoidance^1,4,9^. Feeling anxious about a particular type of thinking, researchers in these literatures hypothesize, should be associated with a tendency to wish to avoid pursuits (e.g., activities, classes, careers) that involve the relevant type of thinking. This hypothesis has a basis in the broader psychology literature, as research in other areas consistently finds that people do, indeed, tend to avoid things they are anxious about. For instance, those who are high in social anxiety tend to avoid social situations^10–12^, and those who are anxious about getting dental work done consistently show a tendency to avoid going to the dentist^13,14^.

While cognition-specific anxieties are consistently predicted to be associated with avoidance of their respective domains, the research base demonstrating this is relatively thin. There is empirical evidence that math-anxious people tend to avoid pursuits that involve math, with work showing that math-anxious individuals tend to avoid courses, majors, and careers that place a large emphasis on doing math^3,15,16^. However, there is little to no work on avoidance tendencies linked to other cognition-specific anxieties, like creativity anxiety and spatial anxiety (feelings of anxiety about having to come up with new ideas or engage in spatial reasoning, respectively). Part of the reason for this lack of research on avoidance in literatures on other cognition-specific anxieties may come down to methodological challenges. A common practice in math anxiety research on avoidance is to designate courses, majors, or careers with the label “math” or “non-math”, or occasionally “STEM” or “non-STEM”, and then ask whether feelings of anxiety about doing math is negatively predictive of whether people tend to engage with pursuits in the math/STEM category^15–18^. This approach works reasonably well for math: indeed, we have used this approach in past work, and every study we know of that has performed such an analysis has found a negative association between math anxiety and engagement with math/STEM pursuits. However, it is not clear how researchers who study other cognition-specific anxieties, like creativity anxiety or spatial anxiety, should determine which pursuits definitively do or do not involve a great deal of the type of thinking they are interested in studying. Creativity anxiety, for instance, is a cognition-specific anxiety we have recently identified that pertains to feelings of anxiety about thinking creatively^1^. A key prediction we have is that high levels of creativity anxiety would be associated with avoidance of creative pursuits, but how exactly should one determine whether a given career is “creative” or not? As we have argued in past work, even quintessentially “creative” domains, like music, do not necessarily require creative thinking^1^. A professional classical violinist may not need to think very creatively at all while performing, instead focusing on playing each of the notes in the precise way they were intended to be played. Labeling “musician” as a creative career, which may have at first seemed like a natural choice, may therefore not be entirely accurate. Additionally, careers that may not seem creative on the surface, like accounting, may often in fact require a great deal of creative thinking (e.g., if a company needs to change their accounting practices in response to a new law). Designating some pursuits as “creative” and other pursuits as “non-creative”, then, seems like a challenge if the goal is to accurately reflect how much creative thinking, in fact, occurs during each type of activity, career, etc. The same issue applies to spatial anxiety and other cognition-specific anxieties: for many domains, there is often no readily agreed-upon list of pursuits that definitively do or do not involve a large amount of the type of thinking in question.

However, from the perspective of someone who is high in, say, creativity anxiety, does it matter how much a given pursuit *actually* involves creative thinking? We contend that, in terms of determining whether someone high in a given cognition-specific anxiety would want to avoid a given pursuit, what is likely to matter more is not the ground truth of how much the relevant type of thinking is actually involved in that pursuit, but rather how much that individual *perceives* that pursuit as involving the type of thinking they are anxious about. Take, for instance, a career that many readers of this paper will be familiar with: “psychologist”. If one wanted to predict whether someone high in creativity anxiety, spatial anxiety, or even math anxiety would be likely to wish to avoid this career, we believe it would be helpful to know how much that person perceived that being a psychologist involves creative thinking, spatial reasoning, or math, respectively. One could imagine two people who are identically high in math anxiety: one who believes that being a psychologist involves a great deal of math (perhaps focusing on the statistical analyses that many psychologists regularly perform) while the other believes that being a psychologist involves very little math (perhaps focusing more on the aspects of the job that involve observing human behavior or theorizing about how the mind works). We would predict that the second person should be more likely to be interested in psychology as a career, since they do not see it as involving much math, a type of thinking they are highly anxious about.

Taking into account individual-level perceptions of the extent to which pursuits involve relevant types of thinking can bring greater nuance to our understanding of links between math anxiety and avoidance of math. Beyond this, though, it can also provide a methodological toehold for studying anxiety-avoidance links involving domains, like creativity and spatial reasoning, that lend themselves less clearly to pursuit categorization than math does. One way to test whether a given cognition-specific anxiety is associated with an avoidance tendency, we contend, is to test whether people high in that cognition-specific anxiety tend to show less interest in pursuits the more they are perceived as involving the type of cognition in question. For instance, if spatial anxiety is, as hypothesized, associated with a tendency to avoid pursuits that involve spatial reasoning, then those high in spatial anxiety should be less interested in pursuits the more they are perceived as involving spatial reasoning. Importantly, this kind of approach bypasses the need for researchers to categorize a priori careers, activities, etc., on the basis of whether *the researchers* believe a given pursuit involves the type of thinking in question, instead placing the focus on *each participant’s* perceptions about these pursuits.

In the present study, we tested whether those higher in cognition-specific anxieties have a tendency to wish to avoid pursuits the more they are perceived as involving the type of thinking that is the target of the anxiety. As an initial test of this hypothesis, we focused on three cognition-specific anxieties: creativity anxiety, math anxiety, and spatial anxiety. Participants separately rated their interest (or lack thereof) in 48 careers and 48 activities^19^. Participants also rated how much they believe each career and activity involves creative thinking, math, and spatial reasoning. Collecting this data allowed us to compute what we call “affinity coefficients”, correlations that reflect intra-individual associations between the perceived involvement of a given type of thinking and interest across pursuits. Taking creativity as an example, if a person had a negative creative affinity coefficient, this means that for that person, the more they perceived a pursuit as involving creative thinking, the less interested they would tend to be in that pursuit. We could then test whether there are unique negative associations between each cognition-specific anxiety and the corresponding affinity coefficient. If this were the case, it would support our core hypothesis that those who feel anxious about a particular type of thinking wish to avoid careers and activities the more they believe they involve the relevant type of thinking. This work allows for additional nuance in our understanding of math anxiety and avoidance by considering differences in the perceived involvement of math across a variety of careers and activities. It also provides, to our knowledge, the first tests of the hypotheses that creativity anxiety and spatial anxiety are associated with avoidance tendencies as well by asking whether those high in these anxieties tend to show less interest in pursuits the more they are perceived as involving creative thinking or spatial reasoning, respectively. Beyond providing insights that are relevant to the literatures about each of these cognitive domains, though, this work has the capacity to provide initial evidence to establish a general principle that feeling anxious about a particular type of thinking is linked to a desire to avoid pursuits that are perceived as involving the type of thinking in question.

## Methods

### Participants

Participants were recruited from two sources: Georgetown University’s undergraduate participant pool and Amazon’s Mechanical Turk (MTurk). The undergraduates who participated were enrolled in at least one psychology course. For the MTurk participants, we required that participants receive the “Master Worker” designation, which limits participation only to MTurk participants who have repeatedly demonstrated that they provide high-quality data on the platform. In total, we collected 333 participants: 157 undergraduate participants and 176 MTurk participants. Two MTurk participants gave the same response to all items and were therefore dropped from analysis, resulting in a final sample of 331 participants (200 female and 131 male; age: *M* = 30.05; *SD* = 11.61; 61.1% Caucasian, 21.3% Asian, 4.8% African or African American, 4.5% Hispanic, 0.6% Native American or Alaskan Native, 7.7% mixed race/other).

### Procedure

All procedures and materials were approved by the Georgetown University Institutional Review Board, and all experimental procedures were performed in accordance with the guidelines and regulations thereof. All participants provided informed consent prior to starting the study. MTurk participants received $4 for participating, and undergraduate participants received course extra credit. Participants completed several questionnaires. The order of all questionnaires was randomized with the exception of a basic demographic questionnaire, which always came at the end of the study. The order of all items within each questionnaire was also randomized. The study was not preregistered.

### Measures

#### Creativity anxiety

Creativity anxiety was measured using the Creativity Anxiety Scale (CAS^1^). The CAS is made up 16 items split evenly split between two types of items: creativity anxiety items, which measure anxiety toward situations that require creative thinking (CA; ex. “Having to think in an open-ended an creative way”) and non-creativity anxiety control items that present similar situations as those in the CA items but remove the need to be creative (NAC; ex. “Having to think in a precise and methodical way”). The NAC items allow for anxiety toward non-creative aspects of the situations presented in the CA items that may be anxiety-inducing (e.g. having to solve a problem) to be measured and statistically controlled for, thereby increasing the specificity of the CA measure. Participants rate how anxious each situation would make them on a scale from 0 (None at all) to 4 (Very much).

#### Math anxiety

Math anxiety was measured using the Single-Item Math Anxiety scale (SIMA^20^) which consists of the single item “On a scale from 1 to 10, how anxious about math are you?”, where 1 was labeled “Not at all anxious” and 10 was labeled “Very anxious”. Note that in the original work on the SIMA, the correlation between SIMA and sMARS (the shortened Math Anxiety Rating Scale, a commonly-used 25-item measure of math anxiety) scores was *r*(277) = 0.77. Here, we used SIMA rather than a measure like sMARS because SIMA was collected from both the undergraduate participants and the MTurk participants while, due to a technical error, sMARS was only collected for the undergraduate participants. SIMA has been shown to have high test–retest reliability (ICC = 0.81^20^).

#### Spatial anxiety

Spatial anxiety was measured using the Spatial Anxiety Scale (SAS^4^). The SAS contains three subscales (Mental Manipulation, Navigation, and Imagery), but the present work is only concerned with the Mental Manipulation subscale, which gauges anxiety toward mentally manipulating objects, as this is the spatial dimension generally thought to be most relevant for most careers^21^. Participants indicated how anxious they would be in various scenarios involving spatial mental manipulation (ex. “Asked to imagine and mentally rotate a 3-dimensional figure”) on a scale from 0 (Not at all) to 4 (Very much). This subscale contains 8 items, with a possible range of scores from 0 to 32 where higher scores indicate greater anxiety. Throughout the present work, we refer to this measure as ‘spatial anxiety’ for simplicity.

#### General trait anxiety

General trait anxiety was measured using the trait subscale of the State-Trait Anxiety Inventory (STAI^22^). Participants responded to 20 items (ex. “I worry too much over something that doesn’t really matter”) meant to assess how often, in general, they experience feelings of anxiety on a scale of 1 (almost never) to 4 (almost always). This measure was included to allow for general tendency to experience anxiety to be controlled for.

#### Career and activity interests

Participants’ interest in a variety of careers and job-related activities was measured using the ‘A’ version of the public domain interest measures developed by Armstrong, Allison, and Rounds^19^. This measure was designed to include items that are representative of a wide array of career and job-related activity types. Participants were shown 48 different careers (ex. “Farmers and Ranchers”; “Biologists”) and asked to rate their level of interest in each on a scale from 1 (Strongly dislike) to 5 (Strongly like). In the activity interest survey, participants were shown 48 different job-related activities (ex. “Direct a play”; “Fix a broken faucet”) and asked to rate their level of interest in that activity using the same response options. A list of all items is shown in the Appendix.

#### Career and activity perceived cognitive involvement

We asked participants to consider each of the 48 careers and 48 activities noted above, and (separately) rate how much they think each involves creative thinking, mathematics, and spatial reasoning. Exact instructions read: “Please indicate how much you think each {career/activity} shown involves Creative Thinking, Spatial Reasoning, and Math.” For each career or activity, the three sub-items (“Involves creative thinking”, “Involves math”, and “Involves spatial reasoning”) were presented in a random order. For each, participants indicated 1 (Not at all) to 5 (Very much) to indicate their perception of how much each career or activity involved that type of thinking.

#### Analytical framework

The core goal of the present work was to understand whether those who report higher levels of anxiety toward specific types of thinking would be more likely to show reduced interest in careers and/or activities to the extent that those pursuits are perceived as involving that particular type of thinking. To address this question for a single career or activity, one could simply examine whether there is an interaction between a given cognition-specific anxiety (e.g., creativity anxiety) and perceived involvement of the specific type of thinking in question (e.g., creative thinking) when predicting interest in that career or activity. The prediction in this case would be that as the perceived cognitive involvement of creative thinking goes up, the (negative) association between creativity anxiety and interest should get stronger. However, such an analysis would afford inferences only about the specific career or activity in question, whereas the goal of this work is to make inferences about careers and job-related activities in general. We therefore sought a framework that would allow us to generalize across pursuits and also respect individual-level variability and nuance in perceptions of pursuits.

Our solution was, for each participant, to compute correlations between their own interest ratings and their own involvement ratings across the 48 careers (and likewise for the 48 activities). We term these intra-individual correlations, ‘affinity coefficients’. For instance, if a specific person is, on average, inclined to show greater interest in a pursuit to the extent that they believe it involves creative thinking, then their creative affinity coefficient should be positive. Namely, there should be a positive correlation, *for that person*, between their interest ratings and their ‘Involves creative thinking’ ratings. That same person might also have a negative affinity coefficient for, say, spatial reasoning, as indicated by a negative correlation between their interest ratings and their ‘Involves spatial reasoning’ ratings. These affinity coefficients, then, would represent the extent to which perceived involvement of particular types of thinking (here, creative thinking, math, or spatial reasoning) are predictive of interest for individual participants. Because affinity coefficients are correlation coefficients, they have possible ranges from − 1 to 1. Note that while the interest measures we collected are typically used to measure interest toward different categories of careers (Holland’s RIASEC categories^23,24^), the goal here was instead to understand intra-individual associations between interest in careers or activities and perceived involvement of specific forms of cognition. To respect what might be important differences in perceptions of careers vs activities, we computed separate affinity coefficients for these two pursuit types, resulting in a total of 6 affinity coefficients (creative, math, and spatial affinity coefficients for both careers and activities).

After computing these affinity coefficients, we could address our main theoretical question by asking whether cognition-specific anxieties negatively predict the affinity coefficients pertaining to the type of thinking in question. For instance, we could ask whether creativity anxiety negatively predicts creative affinity coefficients. If this were the case, it would suggest that as creativity anxiety goes up, the association between perceived involvement of creative thinking and interest for individuals goes down. In the Results below, before addressing this main theoretical question, we first explore whether individuals do, indeed, differ from one another in their perceptions of the types of thinking involved in various careers and activities—a key assumption underlying our desire to consider individual-level perceptions when asking about associations between cognition-specific anxieties and interest in careers and activities.

## Results

### Examining variability in perceptions of how much pursuits involve creative, mathematical, and spatial cognition

Past work provides evidence that there is substantial variability in interest in different careers and activities^19,25^, a pattern we replicate here (Tables 1 and 2). However, no past work, to our knowledge, has examined whether there is substantial variability in the extent to which individual careers and activities are perceived as involving specific forms of cognition. We show the average values for descriptive statistics of interest, and perceived involvement of creative, mathematical, and spatial cognition of all 48 careers and all 48 activities in Tables 1 and 2, respectively.

**Table 1:** Shows the average values for descriptive statistics of ratings for all 48 careers.

Measure	Average values for careers					
	Mean	Median	Mode	SD	Range	Skew
Interest	2.79	2.74	2.73	1.29	4	0.05
Involves creative thinking	3.24	3.28	3.38	1.11	3.98	− 0.11
Involves math	3.08	3.03	3.04	1.09	3.98	0.00
Involves spatial reasoning	3.01	2.98	2.88	1.18	4	0.02

**Table 2:** Shows the average values for descriptive statistics of ratings for all 48 activities.

Measure	Average values for activities					
	Mean	Median	Mode	SD	Range	Skew
Interest	2.90	2.94	2.85	1.27	4	− 0.03
Involves creative thinking	3.19	3.20	2.96	1.10	3.98	− 0.10
Involves math	2.83	2.79	2.56	1.13	4	0.29
Involves spatial reasoning	2.97	2.83	2.65	1.17	4	0.08

**Figure 1:**
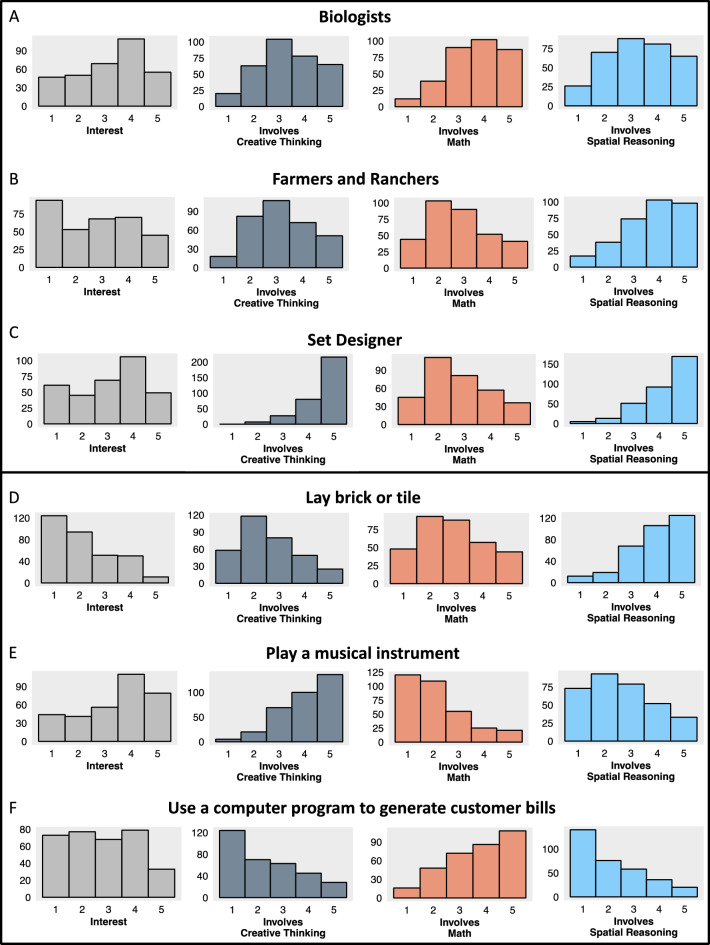
Shows histograms that visualize the distributions of interests and perceived involvement of creative, mathematical, and spatial cognition for example careers (Figure **A**–**C**) and activities (Figure **D**–**F**). Note that the limits of the Y-axis vary from figure to figure to better focus on each distribution.

Results demonstrated substantial variability in perceived involvement of creative, mathematical, and spatial cognition. First, the average standard deviation of all variables is sizeable. In all cases, the coefficient of variation, a metric used to quantify the extent of variability in a measure by computing the ratio of standard deviation to mean^26,27^, is above 0.33. For a useful comparison point, the coefficient of variation for the widely-used measure of trait anxiety employed in this work, the trait component of the State-Trait Anxiety Inventory (STAI^22^), is 0.20 in this sample, and individual differences on this measure are substantial enough to predict a whole host of other measures^28^. The fact that the coefficient of variation is even greater for perceived involvement of various types of thinking across careers and activities suggests that the extent of individual differences in these measures is more than sufficient to allow for associations between these and other variables to be detected (which would not be the case if participants all more or less agreed on how much the careers and activities involved the types of thinking included here). Additionally, the average range on all variables is quite high, at or approaching the maximum of 4 in all cases. In fact, out of the 384 items included (4 variables * 48 careers + 4 variables * 48 activities), the range was the maximum possible for all but 3 items, and in those cases the range was 3. In addition to providing evidence for substantial variability, results from Tables 1 and 2 also indicate that, on average, there are low levels of skewness in these item-level variables. While in the above tables we show averages, see the Appendix for a complete list of descriptive statistics for each item. Additionally, see Fig. 1 for histograms that show the distributions of interests and perceived involvement of creative, mathematical, and spatial cognition for some example careers and activities.

These results demonstrate a considerable degree of variability across individuals in the perceived involvement of specific forms of cognition within individual careers and activities. Different individuals can be asked to consider the same career and have very different ideas of, for example, how much that career involves creative thinking. Taking this variability into account may prove useful in understanding the reasons why individuals differ in their levels of interest in a given pursuit.

### Constructing and exploring the ‘affinity coefficients’

**Figure 2:**
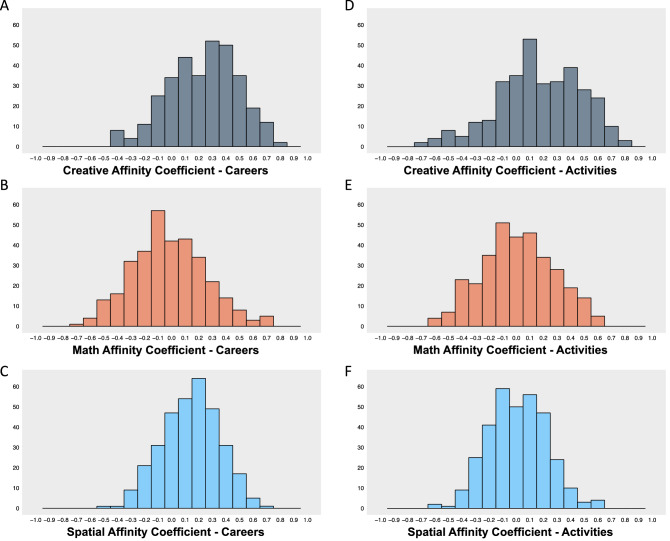
Shows histograms that visualize the distributions of each of the affinity coefficients.

Given that we demonstrated substantial variability in the perceived involvement of different types of thinking in careers and activities, we sought to make use of this variability by constructing the ‘affinity coefficients’ described in the Analytical Approach section of the Methods. For each domain, these affinity coefficients would represent the intra-individual correlation between perceived involvement of a particular type of thinking and interest across all careers or activities. Figure 2 shows the distributions of affinity coefficients for each cognitive domain for both careers and activities. Descriptive statistics are shown in Table 3.

**Table 3:** Shows descriptive statistics for all affinity coefficients.

Measure	Mean	Median	SD	Range	Skew
Creative affinity Coefficient—careers	0.23	0.25	0.26	1.25	− 0.27
Math affinity Coefficient—careers	− 0.02	− 0.03	0.27	1.37	0.20
Spatial affinity Coefficient—careers	0.14	0.15	0.21	1.21	− 0.17
Creative affinity Coefficient—activities	0.17	0.15	0.31	1.53	− 0.32
Math affinity Coefficient—activities	0.01	0.01	0.26	1.22	0.00
Spatial affinity Coefficient—activities	0.01	0.01	0.21	1.22	0.03

These results demonstrate that, as predicted, there is substantial variability in affinity coefficients. This indicates that people do, indeed, differ in the extent to which perceived involvement of specific types of cognition are associated with their interests in both careers and activities. For each of the cognitive domains included here, some individuals are more interested in careers or activities the more that individual perceives them as involving that type of cognition, and others are less interested in them as their perceived involvement of that cognition increases. An important broad point to take from the results thus far is that while interest in careers or activities is associated with a whole host of factors, one of those factors seems to be the perceived involvement of specific types of cognition. In other words, many people seem to care about the sort of thinking they believe is involved in a given pursuit when considering how interested—or not interested—they are in that pursuit.

Having demonstrated substantial variability for each affinity coefficient, we next turn to our main theoretical question. In the next section, we use these coefficients to test whether individuals who have higher levels of a given cognition-specific anxiety also have lower affinity coefficients for that type of cognition.

### Do cognition-specific anxieties predict cognition-specific affinity coefficients?

Having demonstrated substantial variability for each affinity coefficient, we can turn to addressing our main theoretical question: do individuals who have higher levels of a given cognition-specific anxiety also have a tendency to show reduced interest in pursuits (e.g., careers and activities) the more they perceive those pursuits as involving the specific type of thinking that tends to make them feel anxious? To address this idea, in the current section, we test whether we see unique associations between cognition-specific anxieties (creativity anxiety, math anxiety, and spatial anxiety) and affinity coefficients for each respective cognitive domain. If so, these results would suggest that individual differences in perceptions of a given pursuit may be an important heretofore overlooked factor when considering whether an individual high in a given cognition-specific anxiety is likely to want to avoid that pursuit.

**Figure 3:**
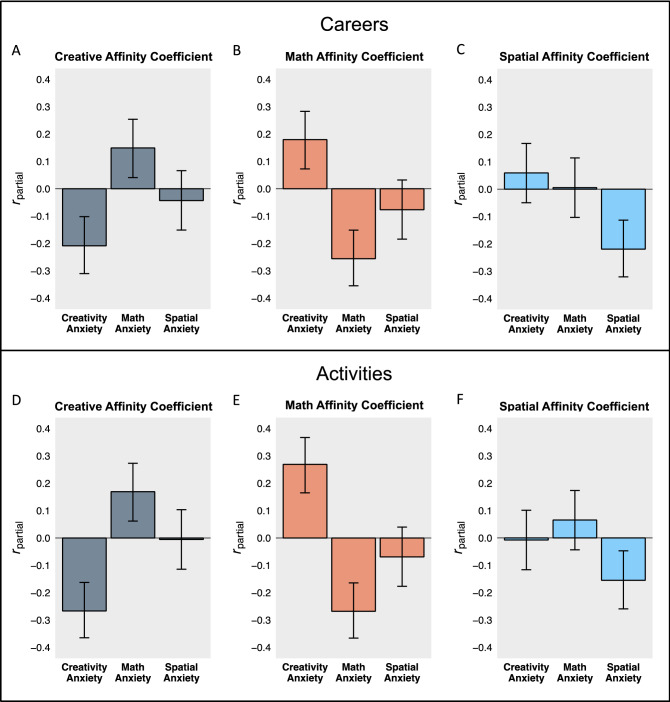
Shows bar plots that reflect partial correlations between cognition-specific anxieties and the affinity coefficients for careers (Fig. 3A–C) and activities (Fig. 3D–F). Each partial correlation controls for the other cognition-specific anxieties in addition to general trait anxiety, non-creativity anxiety control scores, gender, and sample. Error bars indicate 95% confidence intervals.

Our overall prediction was the higher anxiety ratings in a given domain would be related to lower affinity coefficients in that domain (i.e., a *negative* relation), even after controlling for anxiety ratings in the other domains. To use the domain of creativity as an example, we predicted that those who were higher in creativity anxiety would have lower creative affinity coefficients than those lower in creativity anxiety, controlling for both math and spatial anxiety. We ran a separate multiple regression model for each affinity coefficient we computed, resulting in a total of 6 models (each of the 3 domains across both pursuit types, careers and activities). In each model, the key predictors of interest were creativity anxiety, math anxiety, and spatial anxiety. The following variables were included as covariates: general trait anxiety, non-creativity anxiety control scores, gender (female coded as 0, male coded as 1), and a dummy variable indicating which sample the participant was from (the undergraduate sample was coded as 0, and the MTurk sample was coded as 1). See Fig. 3 for a visualization of results for variables of interest expressed as partial Pearson’s correlations. For full model details, see Tables S1 and S2 in Supplementary Materials. Note that, aside from slight differences in estimates, the results presented do not change if the Affinity Coefficients are Fisher z-transformed. The correlations between the untransformed Affinity Coefficients and the corresponding Fisher z-transformed Affinity Coefficients are ≥ 0.995.

Results demonstrate consistent support for our prediction: in every case, whether the pursuit in question is a career or an activity, the affinity coefficient for each domain is significantly negatively associated with the corresponding cognition-specific anxiety (all *p*s < 0.005). For those higher in creativity anxiety, the more they perceive a career or activity as involving creative thinking, the less drawn to it they are; for those higher in math anxiety, the more they perceive a career or activity as involving mathematical thinking, the less drawn to it they are; for those higher in spatial anxiety, the more they perceive a career or activity as involving spatial thinking, the less drawn to it they are.

In parallel to testing our core research question using the approach described above, we additionally tested whether having high levels of a given cognition-specific anxiety is linked to lower interest in pursuits perceived to involve the type of cognition in question using a mixed-effects framework. Specifically, we tested whether there were cross-level interactions between perceived cognitive involvement and cognition-specific anxieties predicting interest (e.g., Creativity Anxiety × Involves Creative Thinking predicting Interest). Results in this framework mirrored the findings presented above: the association between interest and perceived cognitive involvement of each domain depended on the relevant cognition-specific anxiety (all *p*s $\le$ 0.007). Note also that our Affinity Coefficients and the corresponding random slopes between perceived cognitive involvement and interest extracted from these models correlated at *r* = 0.99, suggesting that these approaches largely mirrored one another. For full model details, see Tables S5 and S6.

In addition to treating the affinity coefficients continuously, we also wished to run a follow-up analysis to test whether higher levels of cognition-specific anxieties are associated with a greater likelihood of having a negative affinity coefficient for the domain in question. The previous analysis provided evidence that there is a negative association between cognition-specific anxieties and affinity coefficients within the same form of cognition, but such an association could be observed even if those with high levels of a given cognition-specific anxiety were still drawn to that type of cognition, only less-so than those who are lower in that type of anxiety (i.e., a small positive affinity coefficient as opposed to a large positive affinity coefficient). In this follow-up analysis, we assess whether greater anxiety toward a particular type of thinking is linked to greater odds of wanting to *avoid* pursuits the more they are perceived as including that type of thinking, as indicated by a negative affinity coefficient. Results from logistic regressions using the same variables in the models above find that, in every case, higher values on the relevant cognition-specific anxiety are associated with greater likelihood of having a negative affinity coefficient. A 1 SD increase in creativity anxiety is associated with being 1.67 (*p* = 0.008) times more likely to have a negative creative affinity coefficient for careers and with being 1.83 (*p* = 6E−4) times more likely to have a negative a negative creative affinity coefficient for activities. Additionally, a 1 SD increase in math anxiety is associated with being 1.54 (*p* = 0.001) times more likely to have a negative math affinity coefficient for careers and with being 1.84 (*p* = 1E−5) times more likely to have a negative math affinity coefficient for activities. Finally, a 1 SD increase in spatial anxiety is associated with being 1.40 (*p* = 0.023) times more likely to have a negative spatial affinity coefficient for careers and with being 1.60 (*p* = 0.001) times more likely to have a negative spatial affinity coefficient for activities. See Tables S3 and S4 in Supplementary Materials for full model details (results reported above are in odds ratios, results in these tables are in log-odds). Together, these consistent results indicate that being anxious about a particular type of thinking is linked with a desire to avoid pursuits that are perceived as involving more of that type of thinking.

Thus far, we have only discussed within-domain associations between cognition-specific anxieties and affinity coefficients (e.g., creativity anxiety and creative affinity coefficients). However, in the same models we ran in Fig. 3, we can also examine whether any cross-domain associations exist (e.g., whether creativity anxiety predicts math affinity coefficients). Results when examining cross-domain associations are as consistent as the within-domain results. For the spatial domain, there are no cross-domain associations: spatial anxiety does not predict any affinity coefficient aside from the spatial affinity coefficients, and no cognition-specific anxieties aside from spatial anxiety predict the spatial affinity coefficients. However, in the case of creativity and math, all possible cross-domain associations are significant. Intriguingly, even though creativity anxiety and math anxiety are themselves positively correlated (*r*(329) = 0.184, *p* = 8E−4; controlling for general trait anxiety, *r*_partial_(328) = 0.109, *p* = 0.049), they predict the cross-domain affinity coefficients in the opposite direction from one another: math anxiety is *positively* associated with creative affinity coefficients, and creativity anxiety is *positively* associated with math affinity coefficients. This means that those who are highly anxious about math are more likely to be drawn to pursuits perceived as involving high levels of creative thinking, even after accounting for creativity anxiety, and vice versa.

Together, these results indicate that cognition-specific anxieties are consistently predictive of the strength of intra-individual associations between how much a pursuit (careers or activities) is perceived as involving the relevant form of cognition and interest in that pursuit. Having higher levels of a given cognition-specific anxiety is associated with lower levels of interest in pursuits that are perceived as involving that type of cognition. Additionally, at least in some cases, being high in one type of cognition-specific anxiety (e.g., creativity anxiety) can mean being more interested in pursuits that involve other types of cognition (e.g., math), even after accounting for anxiety toward that other type of cognition.

## Discussion

People who feel anxious about particular types of thinking, like creative thinking, math, or spatial reasoning, are thought to have a tendency to avoid pursuits (e.g., careers or activities) that involve the relevant type of thinking^1,4,9^. However, outside of the literature on math anxiety^3,15,16^, there is very little empirical evidence that this is the case, perhaps because it is more difficult to categorize pursuits as “creative” or “spatial” than it is to categorize them as involving math. In the present work, we developed a framework that bypasses this issue by placing the focus on the perceptions that participants, themselves, hold about how much different careers or activities involve specific types of thinking (like creative thinking). Our hypothesis was that if cognition-specific anxieties are, indeed, associated with avoidance of the relevant type of thinking, then those high in a particular cognition-specific anxiety should have less interest in careers or activities the more they are perceived as involving that type of thinking. To test this hypothesis, we had participants rate their level of interest in a wide variety of different types of careers and activities and also asked them to indicate how much they perceived that each pursuit involved creative thinking, math, and spatial reasoning. This allowed us to generate “affinity coefficients”, within-person associations between the perceived involvement of a type of thinking and interest across pursuits. We predicted that some would have positive affinity coefficients (e.g., the more they perceived pursuits as involving spatial reasoning, the more interested in them they were) while others would have negative affinity coefficients (e.g., the more they perceived pursuits as involving spatial reasoning, the less interested in them they were). We then used these affinity coefficients to test the avoidance hypothesis by asking if cognition-specific anxieties uniquely predict negative affinity coefficients within their respective domain. Our results showed that this was the case in all three domains for both careers and activities, providing consistent evidence that feeling anxious about a type of thinking is linked to a wish to avoid pursuits that are perceived as involving the type of thinking in question. Below, we discuss these findings further, address their limitations, and suggest future directions for this line of work.

The finding that cognition-specific anxieties consistently negatively predict affinity coefficients within their respective domain has several theoretical implications. First, these results provide the first empirical support, to our knowledge, for the idea that creativity anxiety and spatial anxiety are, indeed, associated with tendencies to wish to avoid pursuits that involve creative thinking and spatial reasoning, respectively. This is significant, as avoidance has been hypothesized to be a key consequence of these anxieties^1,4^. Evidence that creativity anxiety and spatial anxiety are linked to a tendency to wish to avoid activities and even careers that are seen as involving these types of thinking suggests that having high levels of these cognition-specific anxieties may be consequential. For one, these avoidance tendencies could contribute to poor cognitive performance by leading people high in creativity anxiety or spatial anxiety to avoid practicing their creative thinking or spatial reasoning, thereby failing to develop these abilities to their full extent. Beyond having implications for how well one might do on a cognitive task that involves the relevant type of thinking, though, evidence that these anxieties are associated with avoidance tendencies suggests that these anxieties could actually, in part, help determine many aspects of one’s life, from the sorts of activities one engages in to the classes one chooses to take to the career one chooses to pursue. While the present research focused on three cognition-specific anxieties as an initial test of the avoidance hypothesis, the consistency of the results suggests that these findings—and the implications we lay out above—have a strong chance of generalizing to other cognitive domains as well.

Additionally, while progress has already been made in demonstrating avoidance tendencies of those high in math anxiety in the past^3,15,16^, the present work can add additional nuance to this understanding. The results presented in this work suggest that the perceptions that individuals, themselves, hold about how much math is involved in pursuits could be an important and previously overlooked factor in determining which types of pursuits math-anxious individuals might wish to avoid. Taking all of the findings of the current work into account, two individuals who are identically high in math anxiety might wish to avoid different types of careers or activities if their perceptions of how much math is involved in careers or activities differs substantially from one another. Thus, this work suggests that current thinking in the math anxiety literature should be updated from “math-anxious individuals tend to avoid pursuits that involve math” to “math-anxious individuals tend to avoid pursuits that they perceive as involving math.” From a methodological standpoint, our results suggest that math anxiety researchers will best be able to predict the sorts of pursuits math-anxious people will avoid by taking into account individual differences in perceived math involvement. This work is also, to our knowledge, some of the first to demonstrate a link between math anxiety and math avoidance as it pertains to specific activities. We found that math-anxious people tend to show less interest in activities the more they are perceived as involving math. This provides important evidence that math-anxious people, may, indeed, engage in so-called “micro-avoidance” behaviors (i.e., avoiding individual situations that involve math) in addition to more “macro-avoidance” behaviors (i.e., not taking elective math courses or pursuing math careers)^15^. This is significant, as it is a tendency to avoid specific situations that involve math that is thought to lead math-anxious people to develop their math ability less over time, partially contributing to their underperformance in math ^9^. While the current work only provides evidence that math-anxious people tend to have less interest in specific activities the more they are perceived as involving math, it can help lay the groundwork for future research that assesses math avoidance behaviors directly.

While the main theoretical goal of this work was to better understand links between cognition-specific anxieties and avoidance, we believe findings related to our “affinity coefficient” framework, also provide some interesting theoretical contributions. In building this approach, we assessed whether people substantially differed from one another in their perceptions of how much a given career or activity involved different types of thinking. Results demonstrated substantial variability in the extent to which participants rated the careers and activities as involving creative thinking, math, and spatial reasoning. Some people, for instance, thought being a biologist involved a great deal of creative thinking, while others thought being a biologist involved very little creative thinking. This variability may owe to the fact that careers are multifaceted in nature and involve many different job functions, so it may be the case that different people focus on different facets of a career when making a judgement of how much a given type of thinking is involved. Interestingly, though, the wide variability we saw in perceptions of the types of thinking involved in careers was also observed in the case of specific job-related activities, even though most of the activities participants rated involved fairly constrained, concrete examples. For instance, when considering the activity “Lay brick or tile”, some believed this activity involved a great deal of math, while others believed it involved very little. These findings provide evidence that people can consider the same career, or even the same specific activity, and come to very different conclusions about how much different types of thinking are involved.

Additionally, we also found substantial individual differences in each of the affinity coefficients we computed. When we observed the distribution of each affinity coefficient, we found that all were normally distributed and centered near zero. This meant that, as we expected, some people had positive affinity coefficients (e.g., the more they thought an activity involved math, the more interested in it they were) while others had negative affinity coefficients (e.g., the more they thought an activity involved math, the less interested in it they were) for each domain and pursuit type. We believe the finding that these affinity coefficients vary so much is meaningful. There are a wide variety of reasons why someone might be interested in, for example, a given career: the sort of impact the career has on the world, the status involved in the career, the pay afforded by a career, etc.^29,30^ The wide variance in affinity coefficients we observed suggests that, particularly for those with strongly negative or strongly positive affinity coefficients, another factor driving interest in careers and activities may be the types of thinking people believe are involved in those pursuits.

### Limitations and future directions

While we believe the current work presents meaningful theoretical and methodological advances, it is not without its limitations. First, our affinity coefficients, while informative, are not measures of observed behavior. As a result, while this work provides evidence of a tendency for those high in the cognition-specific anxieties we measured to wish to avoid pursuits that are perceived as involving the relevant types of thinking, the present work does not provide direct evidence of avoidance *behaviors* linked to these anxieties. Second, while we believe the current work may have implications for cognition-specific anxieties in general, only three were directly studied in the present work: creativity anxiety, math anxiety, and spatial anxiety.

Despite its limitations, we believe the present work can lay the groundwork for significant advances in research on cognition-specific anxieties. For one, the “affinity coefficient” framework is a flexible, easy-to-implement way to study avoidance tendencies associated with other cognition-specific anxieties. Researchers do not have to make a priori assumptions about whether pursuits involve a sufficient amount of the type of thinking in question. Instead, they can simply ask participants themselves to report their own perceptions and use this information alongside interest levels to generate affinity coefficients. In addition to this approach’s flexibility with respect to cognitive domains, it is also flexible with respect to the type of pursuits one wishes to know about. Here, we focused on careers and job-related activities as important pursuits that those high in cognition-specific anxieties may wish to avoid. However, the same affinity coefficient framework could likely just as easily be applied to pursuits like academic courses, majors, extracurricular activities, leisure activities, and so on. And while the present work focused specifically on links between cognition-specific anxieties and avoidance tendencies, we believe the idea behind this framework could be extended to better understand links between anxiety and performance within specific cognitive domains as well. While it is well-documented that those who are anxious about specific types of thinking underperform on tasks that involve the relevant type of thinking ^1,3,4,6,31^, future work could address whether these associations are dependent on individual perceptions of how much a given task involved the type of thinking in question. To use the spatial domain as an example, some people may believe that a computer programming task involves a lot of spatial reasoning while others may believe it involves relatively little. The extent to which spatial anxiety is related to poor performance on the programming task may be a function of the extent to which a given individual believes the task involves spatial reasoning.

Moving forward, researchers could use this affinity coefficient framework, in addition to the general principle that perceptions can shape what those high in cognition-specific anxieties would wish to avoid, to begin building a more comprehensive understanding of links between cognition-specific anxieties and avoidance across a wide array of both anxiety and pursuit types. Doing so, we believe, could allow for a more complete understanding of the circumstances in which people who feel anxious about a particular type of thinking will engage in avoidance.

### Electronic supplementary material

Below is the link to the electronic supplementary material.

Supplementary Tables.

## Data Availability

The data supporting this manuscript can be found at the following Open Science Foundation link: https://osf.io/rqksz/?view_only=235a1ce104574fc69f1e88e40594233b.

## Appendix

### Career descriptives

#### Interest

**Table:**

Career	Mean	Median	Mode	SD	Range	Skew
Farmers and Ranchers	2.75	3.00	1.00	1.42	4.00	0.12
Electronics Engineering Technicians	2.69	3.00	4.00	1.28	4.00	0.07
Fish and Game Wardens	2.44	2.00	1.00	1.32	4.00	0.44
Chemical Technicians	2.56	3.00	1.00	1.23	4.00	0.22
Nuclear Equipment Operation Technicians	2.47	2.00	1.00	1.26	4.00	0.24
Fishery Workers Supervisor	2.18	2.00	1.00	1.16	4.00	0.60
Petroleum Engineers	2.38	2.00	1.00	1.26	4.00	0.46
Civil Engineers	2.89	3.00	4.00	1.28	4.00	− 0.07
Biochemists	2.87	3.00	4.00	1.32	4.00	− 0.08
Dentists, General	2.55	2.00	1.00	1.32	4.00	0.27
Veterinarians	3.37	4.00	4.00	1.33	4.00	− 0.49
Biologists	3.23	3.00	4.00	1.29	4.00	− 0.36
Epidemiologists	3.03	3.00	4.00	1.20	4.00	− 0.28
Surgeons	3.14	3.00	4.00	1.44	4.00	− 0.21
Orthodontists	2.58	2.00	1.00	1.29	4.00	0.23
Animal Scientists	3.38	4.00	4.00	1.26	4.00	− 0.56
Musicians, Instrumental	3.17	3.00	4.00	1.44	4.00	− 0.30
Professional Photographers	3.66	4.00	4.00	1.20	4.00	− 0.83
Singers	3.02	3.00	4.00	1.47	4.00	− 0.12
English Language College Teachers	3.04	3.00	4.00	1.38	4.00	− 0.15
Art, Drama, and Music College Teachers	2.90	3.00	1.00	1.46	4.00	− 0.01
Set Designers	3.11	3.00	4.00	1.34	4.00	− 0.30
Curators	3.08	3.00	4.00	1.26	4.00	− 0.26
Music Directors	2.85	3.00	4.00	1.39	4.00	0.02
Physical Therapist Aides	3.08	3.00	4.00	1.23	4.00	− 0.32
Mental Health Counselors	3.48	4.00	4.00	1.38	4.00	− 0.56
Athletic Trainers	3.07	3.00	4.00	1.28	4.00	− 0.22
Child Care Workers	3.03	3.00	4.00	1.35	4.00	− 0.17
Secondary School Teachers	3.12	3.00	4.00	1.36	4.00	− 0.30
Personal and Home Care Aides	2.74	3.00	4.00	1.29	4.00	0.08
Speech Language Pathologists	3.07	3.00	4.00	1.31	4.00	− 0.24
Middle School Teachers	2.94	3.00	4.00	1.38	4.00	− 0.02
Purchasing Managers	2.92	3.00	3.00	1.19	4.00	− 0.10
Sales Agents, Financial Services	2.62	3.00	2.00	1.27	4.00	0.28
Food Service Managers	2.55	2.00	2.00	1.22	4.00	0.32
Telemarketers	1.64	1.00	1.00	1.04	4.00	1.65
Retail Salesperson	2.45	2.00	2.00	1.17	4.00	0.36
Insurance Sales Agents	2.14	2.00	1.00	1.15	4.00	0.81
Lawyers	3.22	4.00	4.00	1.30	4.00	− 0.31
Real Estate Sales Agents	2.88	3.00	4.00	1.29	4.00	− 0.02
Auditors	2.45	2.00	1.00	1.28	4.00	0.36
Payroll and Timekeeping Clerks	2.44	2.00	1.00	1.25	4.00	0.36
Shipping and Receiving Clerks	2.49	2.50	1.00	1.23	4.00	0.22
Meter Readers, Utilities	2.20	2.00	1.00	1.22	4.00	0.58
Accountants	2.59	2.00	1.00	1.35	4.00	0.26
Mail Clerks	2.46	2.00	1.00	1.26	4.00	0.34
Actuaries	2.48	3.00	3.00	1.12	4.00	0.16
Tellers	2.48	2.00	2.00	1.21	4.00	0.34

#### Involves creative thinking

**Table:**

Career	Mean	Median	Mode	SD	Range	Skew
Farmers and Ranchers	3.17	3.00	3.00	1.13	4.00	0.08
Electronics Engineering Technicians	3.42	3.00	5.00	1.28	4.00	− 0.30
Fish and Game Wardens	2.68	3.00	3.00	1.19	4.00	0.33
Chemical Technicians	2.93	3.00	2.00	1.30	4.00	0.16
Nuclear Equipment Operation Technicians	3.20	3.00	3.00	1.37	4.00	− 0.05
Fishery Workers Supervisor	2.45	2.00	2.00	1.11	4.00	0.46
Petroleum Engineers	3.04	3.00	3.00	1.24	4.00	− 0.02
Civil Engineers	3.75	4.00	5.00	1.14	4.00	− 0.56
Biochemists	3.30	3.00	3.00	1.24	4.00	− 0.14
Dentists, General	2.77	3.00	2.00	1.18	4.00	0.32
Veterinarians	3.22	3.00	3.00	1.12	4.00	− 0.08
Biologists	3.32	3.00	3.00	1.17	4.00	− 0.12
Epidemiologists	3.20	3.00	3.00	1.22	4.00	− 0.02
Surgeons	3.65	4.00	5.00	1.19	4.00	− 0.51
Orthodontists	2.77	3.00	3.00	1.15	4.00	0.27
Animal Scientists	3.23	3.00	3.00	1.17	4.00	− 0.01
Musicians, Instrumental	4.32	5.00	5.00	0.91	4.00	− 1.20
Professional Photographers	4.44	5.00	5.00	0.80	4.00	− 1.41
Singers	4.16	5.00	5.00	1.07	4.00	− 1.23
English Language College Teachers	3.88	4.00	5.00	1.02	4.00	− 0.64
Art, Drama, and Music College Teachers	4.50	5.00	5.00	0.79	4.00	− 1.57
Set Designers	4.53	5.00	5.00	0.74	3.00	− 1.52
Curators	3.69	4.00	5.00	1.14	4.00	− 0.52
Music Directors	4.33	5.00	5.00	0.89	4.00	− 1.23
Physical Therapist Aides	3.12	3.00	3.00	1.11	4.00	0.05
Mental Health Counselors	3.86	4.00	5.00	1.02	4.00	− 0.50
Athletic Trainers	3.29	3.00	3.00	1.11	4.00	− 0.17
Child Care Workers	3.93	4.00	5.00	1.05	4.00	− 0.60
Secondary School Teachers	3.98	4.00	5.00	0.93	4.00	− 0.48
Personal and Home Care Aides	3.14	3.00	3.00	1.10	4.00	0.11
Speech Language Pathologists	3.65	4.00	4.00	1.00	4.00	− 0.40
Middle School Teachers	3.94	4.00	5.00	0.97	4.00	− 0.52
Purchasing Managers	2.84	3.00	3.00	1.16	4.00	0.19
Sales Agents, Financial Services	3.14	3.00	3.00	1.17	4.00	0.04
Food Service Managers	2.80	3.00	3.00	1.18	4.00	0.26
Telemarketers	3.06	3.00	3.00	1.35	4.00	− 0.04
Retail Salesperson	3.09	3.00	3.00	1.21	4.00	0.05
Insurance Sales Agents	2.94	3.00	3.00	1.20	4.00	0.15
Lawyers	3.96	4.00	5.00	1.04	4.00	− 0.74
Real Estate Sales Agents	3.48	4.00	4.00	1.05	4.00	− 0.26
Auditors	2.36	2.00	2.00	1.22	4.00	0.69
Payroll and Timekeeping Clerks	1.95	2.00	1.00	1.10	4.00	1.10
Shipping and Receiving Clerks	2.15	2.00	1.00	1.17	4.00	0.82
Meter Readers, Utilities	1.84	1.00	1.00	1.08	4.00	1.20
Accountants	2.36	2.00	2.00	1.22	4.00	0.75
Mail Clerks	1.95	2.00	1.00	1.06	4.00	1.14
Actuaries	2.59	2.50	2.00	1.19	4.00	0.39
Tellers	2.08	2.00	1.00	1.16	4.00	1.00

#### Involves math

**Table:**

Career	Mean	Median	Mode	SD	Range	Skew
Farmers and Ranchers	2.83	3.00	2.00	1.21	4.00	0.30
Electronics Engineering Technicians	4.38	5.00	5.00	0.97	4.00	− 1.68
Fish and Game Wardens	2.33	2.00	2.00	1.11	4.00	0.67
Chemical Technicians	4.14	4.00	5.00	0.95	4.00	− 0.98
Nuclear Equipment Operation Technicians	4.30	5.00	5.00	0.98	4.00	− 1.31
Fishery Workers Supervisor	2.65	2.00	2.00	1.14	4.00	0.41
Petroleum Engineers	4.23	4.50	5.00	0.94	4.00	− 1.17
Civil Engineers	4.46	5.00	5.00	0.87	4.00	− 1.75
Biochemists	4.12	4.00	5.00	0.99	4.00	− 0.95
Dentists, General	2.76	3.00	2.00	1.15	4.00	0.35
Veterinarians	2.92	3.00	3.00	1.18	4.00	0.21
Biologists	3.65	4.00	4.00	1.10	4.00	− 0.45
Epidemiologists	3.43	3.00	3.00	1.23	4.00	− 0.31
Surgeons	3.42	3.00	3.00	1.22	4.00	− 0.21
Orthodontists	2.76	3.00	3.00	1.19	4.00	0.26
Animal Scientists	3.17	3.00	3.00	1.22	4.00	− 0.08
Musicians, Instrumental	2.24	2.00	2.00	1.18	4.00	0.86
Professional Photographers	2.17	2.00	2.00	1.11	4.00	0.87
Singers	1.68	1.00	1.00	1.01	4.00	1.70
English Language College Teachers	1.75	1.00	1.00	1.06	4.00	1.60
Art, Drama, and Music College Teachers	2.12	2.00	1.00	1.16	4.00	0.99
Set Designers	2.78	3.00	2.00	1.20	4.00	0.33
Curators	2.33	2.00	2.00	1.07	4.00	0.59
Music Directors	2.15	2.00	2.00	1.12	4.00	0.89
Physical Therapist Aides	2.28	2.00	2.00	1.04	4.00	0.72
Mental Health Counselors	1.94	2.00	1.00	1.06	4.00	1.08
Athletic Trainers	2.33	2.00	2.00	1.15	4.00	0.71
Child Care Workers	2.03	2.00	2.00	1.02	4.00	1.04
Secondary School Teachers	3.42	3.00	3.00	0.97	4.00	0.10
Personal and Home Care Aides	2.04	2.00	2.00	1.05	4.00	0.97
Speech Language Pathologists	2.00	2.00	1.00	1.09	4.00	1.02
Middle School Teachers	3.53	3.00	3.00	1.01	4.00	0.00
Purchasing Managers	3.98	4.00	5.00	0.99	4.00	− 0.72
Sales Agents, Financial Services	4.13	4.00	5.00	0.95	4.00	− 0.96
Food Service Managers	2.86	3.00	3.00	1.17	4.00	0.12
Telemarketers	2.16	2.00	1.00	1.18	4.00	0.79
Retail Salesperson	3.08	3.00	3.00	1.21	4.00	0.15
Insurance Sales Agents	3.74	4.00	5.00	1.14	4.00	− 0.54
Lawyers	2.48	2.00	2.00	1.12	4.00	0.49
Real Estate Sales Agents	3.38	3.00	3.00	1.10	4.00	− 0.17
Auditors	4.19	5.00	5.00	1.15	4.00	− 1.31
Payroll and Timekeeping Clerks	4.19	5.00	5.00	1.01	4.00	− 1.07
Shipping and Receiving Clerks	3.23	3.00	3.00	1.16	4.00	0.00
Meter Readers, Utilities	3.23	3.00	3.00	1.19	4.00	0.01
Accountants	4.73	5.00	5.00	0.62	3.00	− 2.53
Mail Clerks	2.32	2.00	2.00	1.12	4.00	0.74
Actuaries	3.90	4.00	5.00	1.22	4.00	− 0.73
Tellers	4.00	4.00	5.00	1.10	4.00	− 0.94

#### Involves spatial reasoning

**Table:**

Career	Mean	Median	Mode	SD	Range	Skew
Farmers and Ranchers	3.69	4.00	4.00	1.16	4.00	− 0.60
Electronics Engineering Technicians	3.80	4.00	4.00	1.11	4.00	− 0.67
Fish and Game Wardens	3.02	3.00	3.00	1.21	4.00	− 0.01
Chemical Technicians	3.22	3.00	4.00	1.25	4.00	− 0.16
Nuclear Equipment Operation Technicians	3.85	4.00	5.00	1.11	4.00	− 0.65
Fishery Workers Supervisor	2.87	3.00	3.00	1.17	4.00	0.17
Petroleum Engineers	3.60	4.00	4.00	1.10	4.00	− 0.36
Civil Engineers	4.25	5.00	5.00	0.96	4.00	− 1.31
Biochemists	3.32	3.00	4.00	1.23	4.00	− 0.30
Dentists, General	3.63	4.00	4.00	1.06	4.00	− 0.51
Veterinarians	3.14	3.00	3.00	1.19	4.00	0.06
Biologists	3.27	3.00	3.00	1.22	4.00	− 0.13
Epidemiologists	3.09	3.00	3.00	1.24	4.00	− 0.01
Surgeons	4.28	5.00	5.00	0.95	4.00	− 1.36
Orthodontists	3.67	4.00	4.00	1.12	4.00	− 0.58
Animal Scientists	3.17	3.00	3.00	1.20	4.00	− 0.13
Musicians, Instrumental	2.78	3.00	2.00	1.23	4.00	0.23
Professional Photographers	4.03	4.00	5.00	1.08	4.00	− 0.87
Singers	2.25	2.00	1.00	1.18	4.00	0.62
English Language College Teachers	2.32	2.00	1.00	1.26	4.00	0.66
Art, Drama, and Music College Teachers	3.28	3.00	3.00	1.24	4.00	− 0.08
Set Designers	4.23	5.00	5.00	0.95	4.00	− 1.17
Curators	3.39	4.00	4.00	1.21	4.00	− 0.32
Music Directors	2.88	3.00	3.00	1.23	4.00	0.17
Physical Therapist Aides	3.42	3.50	4.00	1.11	4.00	− 0.29
Mental Health Counselors	2.55	2.00	1.00	1.35	4.00	0.38
Athletic Trainers	3.35	3.00	3.00	1.17	4.00	− 0.19
Child Care Workers	2.96	3.00	3.00	1.24	4.00	0.09
Secondary School Teachers	3.07	3.00	3.00	1.20	4.00	0.11
Personal and Home Care Aides	2.90	3.00	3.00	1.16	4.00	0.10
Speech Language Pathologists	2.55	2.50	3.00	1.25	4.00	0.33
Middle School Teachers	3.06	3.00	3.00	1.20	4.00	0.07
Purchasing Managers	2.65	3.00	3.00	1.17	4.00	0.38
Sales Agents, Financial Services	2.40	2.00	2.00	1.21	4.00	0.54
Food Service Managers	2.69	3.00	3.00	1.15	4.00	0.19
Telemarketers	1.92	1.00	1.00	1.20	4.00	1.06
Retail Salesperson	2.64	2.00	2.00	1.20	4.00	0.39
Insurance Sales Agents	2.28	2.00	1.00	1.24	4.00	0.71
Lawyers	2.63	2.00	1.00	1.34	4.00	0.36
Real Estate Sales Agents	3.25	3.00	3.00	1.18	4.00	− 0.23
Auditors	2.36	2.00	1.00	1.24	4.00	0.65
Payroll and Timekeeping Clerks	2.15	2.00	1.00	1.18	4.00	0.77
Shipping and Receiving Clerks	2.91	3.00	3.00	1.22	4.00	0.06
Meter Readers, Utilities	2.42	2.00	2.00	1.16	4.00	0.47
Accountants	2.28	2.00	1.00	1.28	4.00	0.75
Mail Clerks	2.64	3.00	2.00	1.17	4.00	0.29
Actuaries	2.46	2.00	3.00	1.18	4.00	0.42
Tellers	2.08	2.00	1.00	1.15	4.00	0.89

### Activity descriptives

#### Interest

**Table:**

Activity	Mean	Median	Mode	SD	Range	Skew
Test the quality of parts before shipment	2.55	2.00	1.00	1.28	4.00	0.28
Lay brick or tile	2.18	2.00	1.00	1.19	4.00	0.67
Work on an offshore oil-drilling rig	1.76	1.00	1.00	1.11	4.00	1.29
Assemble electronic parts	2.58	2.00	1.00	1.31	4.00	0.29
Operate a grinding machine in a factory	1.94	1.00	1.00	1.17	4.00	1.04
Fix a broken faucet	2.30	2.00	1.00	1.22	4.00	0.59
Assemble products in a factory	2.26	2.00	1.00	1.26	4.00	0.61
Install flooring in houses	2.19	2.00	1.00	1.21	4.00	0.74
Study the structure of the human body	3.47	4.00	4.00	1.23	4.00	− 0.62
Study animal behavior	3.70	4.00	4.00	1.18	4.00	− 0.80
Do research on plants or animals	3.41	4.00	4.00	1.26	4.00	− 0.54
Develop a new medical treatment or procedure	3.39	4.00	4.00	1.27	4.00	− 0.45
Conduct biological research	3.26	4.00	4.00	1.34	4.00	− 0.41
Study whales and other types of marine life	3.53	4.00	4.00	1.21	4.00	− 0.66
Work in a biology lab	3.15	3.00	4.00	1.29	4.00	− 0.30
Make a map of the bottom of an ocean	2.84	3.00	4.00	1.25	4.00	0.01
Conduct a Musical Choir	2.47	2.00	1.00	1.30	4.00	0.43
Direct a Play	2.65	2.00	1.00	1.41	4.00	0.34
Design Artwork for Magazines	3.14	4.00	4.00	1.39	4.00	− 0.34
Write a Song	2.91	3.00	4.00	1.44	4.00	− 0.02
Write Books or Plays	3.19	4.00	4.00	1.43	4.00	− 0.25
Play a Musical Instrument	3.42	4.00	4.00	1.33	4.00	− 0.53
Perform Stunts for a movie or television show	2.44	2.00	1.00	1.36	4.00	0.39
Design sets for plays	3.11	3.00	4.00	1.35	4.00	− 0.21
Give career guidance to people	3.30	4.00	4.00	1.28	4.00	− 0.43
Do volunteer work at a non-profit organization	3.85	4.00	4.00	1.12	4.00	− 0.98
Help people who have problems with drugs or alcohol	3.17	4.00	4.00	1.34	4.00	− 0.35
Teach an individual an exercise routine	3.20	3.00	4.00	1.27	4.00	− 0.33
Help people with family-related problems	3.41	4.00	4.00	1.29	4.00	− 0.56
Supervise the activities of children at a camp	3.27	4.00	4.00	1.33	4.00	− 0.40
Teach children how to read	3.52	4.00	4.00	1.27	4.00	− 0.60
Help elderly people with their daily activities	3.02	3.00	4.00	1.27	4.00	− 0.20
Sell restaurant franchises to individuals	2.48	2.00	1.00	1.22	4.00	0.30
Sell merchandise at a department store	2.46	2.00	2.00	1.17	4.00	0.32
Manage the operations of a hotel	3.11	3.00	4.00	1.19	4.00	− 0.23
Operate a beauty salon or barber shop	2.36	2.00	1.00	1.19	4.00	0.43
Manage a department within a large company	3.35	4.00	4.00	1.28	4.00	− 0.47
Manage a clothing store	2.92	3.00	4.00	1.27	4.00	− 0.12
Sell houses	2.82	3.00	4.00	1.29	4.00	0.01
Run a toy store	3.05	3.00	4.00	1.27	4.00	− 0.26
Generate the monthly payroll checks for an office	2.59	2.00	1.00	1.32	4.00	0.29
Inventory supplies using a hand-held computer	2.56	2.00	1.00	1.31	4.00	0.25
Use a computer program to generate customer bills	2.76	3.00	4.00	1.31	4.00	0.11
Maintain employee records	2.76	3.00	3.00	1.28	4.00	0.15
Compute and record statistical and other numerical data	2.97	3.00	4.00	1.37	4.00	− 0.09
Operate a calculator	3.23	3.00	3.00	1.18	4.00	− 0.27
Handle customers' bank transactions	2.64	3.00	1.00	1.30	4.00	0.22
Keep shipping and receiving records	2.50	2.00	1.00	1.28	4.00	0.31

#### Involves creative thinking

**Table:**

Activity	Mean	Median	Mode	SD	Range	Skew
Test the quality of parts before shipment	2.35	2.00	1.00	1.22	4.00	0.60
Lay brick or tile	2.59	2.00	2.00	1.16	4.00	0.45
Work on an offshore oil-drilling rig	2.32	2.00	1.00	1.19	4.00	0.62
Assemble electronic parts	2.68	3.00	2.00	1.24	4.00	0.31
Operate a grinding machine in a factory	1.92	2.00	1.00	1.12	4.00	1.14
Fix a broken faucet	2.71	3.00	2.00	1.13	4.00	0.29
Assemble products in a factory	2.14	2.00	1.00	1.23	4.00	0.84
Install flooring in houses	2.86	3.00	2.00	1.18	4.00	0.26
Study the structure of the human body	2.66	2.00	2.00	1.30	4.00	0.37
Study animal behavior	3.15	3.00	3.00	1.20	4.00	− 0.05
Do research on plants or animals	3.20	3.00	2.00	1.19	4.00	− 0.02
Develop a new medical treatment or procedure	4.22	5.00	5.00	0.97	4.00	− 0.99
Conduct biological research	3.46	4.00	4.00	1.17	4.00	− 0.27
Study whales and other types of marine life	3.07	3.00	2.00	1.17	4.00	0.07
Work in a biology lab	3.25	3.00	3.00	1.20	4.00	0.01
Make a map of the bottom of an ocean	3.26	3.00	3.00	1.29	4.00	0.00
Conduct a Musical Choir	3.97	4.00	5.00	1.08	4.00	− 0.77
Direct a Play	4.48	5.00	5.00	0.85	4.00	− 1.74
Design Artwork for Magazines	4.60	5.00	5.00	0.75	3.00	− 1.96
Write a Song	4.67	5.00	5.00	0.71	4.00	− 2.47
Write Books or Plays	4.70	5.00	5.00	0.68	4.00	− 2.62
Play a Musical Instrument	4.04	4.00	5.00	1.00	4.00	− 0.80
Perform Stunts for a movie or television show	3.52	4.00	4.00	1.19	4.00	− 0.39
Design sets for plays	4.45	5.00	5.00	0.81	4.00	− 1.41
Give career guidance to people	3.89	4.00	4.00	0.98	4.00	− 0.50
Do volunteer work at a non-profit organization	3.11	3.00	3.00	1.21	4.00	− 0.03
Help people who have problems with drugs or alcohol	3.81	4.00	5.00	1.07	4.00	− 0.63
Teach an individual an exercise routine	3.28	3.00	3.00	1.09	4.00	− 0.02
Help people with family-related problems	3.90	4.00	5.00	1.02	4.00	− 0.61
Supervise the activities of children at a camp	3.97	4.00	5.00	1.03	4.00	− 0.76
Teach children how to read	3.85	4.00	5.00	1.06	4.00	− 0.54
Help elderly people with their daily activities	3.24	3.00	3.00	1.14	4.00	− 0.06
Sell restaurant franchises to individuals	3.44	4.00	4.00	1.14	4.00	− 0.36
Sell merchandise at a department store	3.16	3.00	3.00	1.20	4.00	0.05
Manage the operations of a hotel	3.47	3.00	3.00	1.11	4.00	− 0.15
Operate a beauty salon or barber shop	3.55	4.00	4.00	1.09	4.00	− 0.35
Manage a department within a large company	3.53	4.00	3.00	1.07	4.00	− 0.18
Manage a clothing store	3.38	3.00	3.00	1.09	4.00	− 0.08
Sell houses	3.59	4.00	3.00	1.08	4.00	− 0.34
Run a toy store	3.52	3.00	3.00	1.09	4.00	− 0.20
Generate the monthly payroll checks for an office	1.89	1.50	1.00	1.13	4.00	1.21
Inventory supplies using a hand-held computer	1.96	2.00	1.00	1.08	4.00	1.09
Use a computer program to generate customer bills	2.34	2.00	1.00	1.33	4.00	0.58
Maintain employee records	2.00	2.00	1.00	1.11	4.00	0.99
Compute and record statistical and other numerical data	2.26	2.00	1.00	1.24	4.00	0.76
Operate a calculator	1.79	1.00	1.00	1.09	4.00	1.44
Handle customers' bank transactions	2.04	2.00	1.00	1.15	4.00	1.08
Keep shipping and receiving records	1.97	2.00	1.00	1.16	4.00	1.13

#### Involves math

**Table:**

Activity	Mean	Median	Mode	SD	Range	Skew
Test the quality of parts before shipment	2.65	2.00	2.00	1.27	4.00	0.38
Lay brick or tile	2.87	3.00	2.00	1.25	4.00	0.21
Work on an offshore oil-drilling rig	2.82	3.00	2.00	1.25	4.00	0.27
Assemble electronic parts	2.88	3.00	3.00	1.28	4.00	0.10
Operate a grinding machine in a factory	2.22	2.00	1.00	1.22	4.00	0.79
Fix a broken faucet	2.17	2.00	2.00	1.11	4.00	0.84
Assemble products in a factory	2.45	2.00	2.00	1.26	4.00	0.60
Install flooring in houses	3.38	3.00	3.00	1.23	4.00	− 0.18
Study the structure of the human body	2.74	3.00	2.00	1.28	4.00	0.36
Study animal behavior	2.49	2.00	2.00	1.21	4.00	0.58
Do research on plants or animals	3.15	3.00	3.00	1.18	4.00	− 0.02
Develop a new medical treatment or procedure	3.75	4.00	5.00	1.18	4.00	− 0.63
Conduct biological research	3.61	4.00	3.00	1.11	4.00	− 0.32
Study whales and other types of marine life	2.73	3.00	2.00	1.15	4.00	0.28
Work in a biology lab	3.66	4.00	4.00	1.12	4.00	− 0.48
Make a map of the bottom of an ocean	3.68	4.00	5.00	1.18	4.00	− 0.52
Conduct a Musical Choir	2.05	2.00	2.00	1.10	4.00	1.10
Direct a Play	1.80	1.00	1.00	1.03	4.00	1.38
Design Artwork for Magazines	2.08	2.00	1.00	1.09	4.00	0.92
Write a Song	1.75	1.00	1.00	0.97	4.00	1.45
Write Books or Plays	1.62	1.00	1.00	0.93	4.00	1.69
Play a Musical Instrument	2.15	2.00	1.00	1.18	4.00	0.93
Perform Stunts for a movie or television show	2.16	2.00	1.00	1.21	4.00	0.88
Design sets for plays	2.73	3.00	2.00	1.20	4.00	0.31
Give career guidance to people	2.15	2.00	2.00	1.10	4.00	0.97
Do volunteer work at a non-profit organization	2.15	2.00	2.00	1.07	4.00	0.84
Help people who have problems with drugs or alcohol	1.76	1.00	1.00	1.01	4.00	1.48
Teach an individual an exercise routine	1.94	2.00	1.00	1.09	4.00	1.18
Help people with family-related problems	1.65	1.00	1.00	0.96	4.00	1.63
Supervise the activities of children at a camp	1.96	2.00	1.00	1.08	4.00	1.23
Teach children how to read	1.62	1.00	1.00	1.04	4.00	1.72
Help elderly people with their daily activities	1.87	2.00	1.00	1.02	4.00	1.25
Sell restaurant franchises to individuals	3.43	3.00	3.00	1.18	4.00	− 0.29
Sell merchandise at a department store	2.88	3.00	2.00	1.20	4.00	0.34
Manage the operations of a hotel	3.63	4.00	4.00	1.10	4.00	− 0.38
Operate a beauty salon or barber shop	2.95	3.00	2.00	1.21	4.00	0.16
Manage a department within a large company	3.46	4.00	4.00	1.13	4.00	− 0.28
Manage a clothing store	3.30	3.00	3.00	1.14	4.00	− 0.09
Sell houses	3.41	3.00	4.00	1.16	4.00	− 0.20
Run a toy store	3.34	3.00	3.00	1.12	4.00	− 0.16
Generate the monthly payroll checks for an office	4.20	5.00	5.00	1.00	4.00	− 1.03
Inventory supplies using a hand-held computer	3.31	3.00	2.00	1.23	4.00	− 0.06
Use a computer program to generate customer bills	3.67	4.00	5.00	1.21	4.00	− 0.51
Maintain employee records	3.19	3.00	3.00	1.23	4.00	0.01
Compute and record statistical and other numerical data	4.61	5.00	5.00	0.73	4.00	− 2.10
Operate a calculator	4.09	5.00	5.00	1.16	4.00	− 1.02
Handle customers' bank transactions	4.30	5.00	5.00	0.96	4.00	− 1.25
Keep shipping and receiving records	3.58	4.00	5.00	1.19	4.00	− 0.35

#### Involves spatial reasoning

**Table:**

Activity	Mean	Median	Mode	SD	Range	Skew
Test the quality of parts before shipment	2.98	3.00	3.00	1.19	4.00	0.05
Lay brick or tile	3.95	4.00	5.00	1.07	4.00	− 0.89
Work on an offshore oil-drilling rig	3.41	3.00	4.00	1.22	4.00	− 0.30
Assemble electronic parts	3.77	4.00	4.00	1.08	4.00	− 0.59
Operate a grinding machine in a factory	3.07	3.00	3.00	1.17	4.00	− 0.03
Fix a broken faucet	3.36	3.00	3.00	1.12	4.00	− 0.18
Assemble products in a factory	3.44	3.00	3.00	1.17	4.00	− 0.30
Install flooring in houses	4.11	4.00	5.00	0.99	4.00	− 0.83
Study the structure of the human body	3.82	4.00	4.00	1.09	4.00	− 0.67
Study animal behavior	3.03	3.00	3.00	1.15	4.00	0.02
Do research on plants or animals	3.10	3.00	3.00	1.14	4.00	0.00
Develop a new medical treatment or procedure	3.32	3.00	3.00	1.29	4.00	− 0.23
Conduct biological research	3.16	3.00	3.00	1.21	4.00	− 0.07
Study whales and other types of marine life	3.18	3.00	3.00	1.16	4.00	− 0.10
Work in a biology lab	3.23	3.00	3.00	1.16	4.00	− 0.06
Make a map of the bottom of an ocean	4.38	5.00	5.00	0.91	4.00	− 1.48
Conduct a Musical Choir	2.79	3.00	3.00	1.20	4.00	0.24
Direct a Play	3.58	4.00	4.00	1.16	4.00	− 0.44
Design Artwork for Magazines	3.57	4.00	4.00	1.16	4.00	− 0.40
Write a Song	1.94	2.00	1.00	1.11	4.00	1.03
Write Books or Plays	2.41	2.00	1.00	1.31	4.00	0.57
Play a Musical Instrument	2.63	2.00	2.00	1.26	4.00	0.35
Perform Stunts for a movie or television show	3.95	4.00	5.00	1.14	4.00	− 0.82
Design sets for plays	4.14	4.00	5.00	1.02	4.00	− 1.16
Give career guidance to people	2.26	2.00	1.00	1.28	4.00	0.68
Do volunteer work at a non-profit organization	2.52	2.00	2.00	1.12	4.00	0.43
Help people who have problems with drugs or alcohol	2.16	2.00	1.00	1.24	4.00	0.84
Teach an individual an exercise routine	3.19	3.00	3.00	1.15	4.00	− 0.10
Help people with family-related problems	2.16	2.00	1.00	1.25	4.00	0.84
Supervise the activities of children at a camp	3.12	3.00	3.00	1.20	4.00	− 0.09
Teach children how to read	2.28	2.00	1.00	1.20	4.00	0.67
Help elderly people with their daily activities	2.93	3.00	3.00	1.18	4.00	0.13
Sell restaurant franchises to individuals	2.57	2.00	2.00	1.23	4.00	0.30
Sell merchandise at a department store	2.63	2.00	2.00	1.23	4.00	0.37
Manage the operations of a hotel	3.16	3.00	3.00	1.19	4.00	− 0.04
Operate a beauty salon or barber shop	2.96	3.00	3.00	1.12	4.00	0.17
Manage a department within a large company	2.93	3.00	2.00	1.23	4.00	0.07
Manage a clothing store	3.23	3.00	3.00	1.11	4.00	− 0.09
Sell houses	3.11	3.00	3.00	1.14	4.00	− 0.14
Run a toy store	3.25	3.00	3.00	1.08	4.00	− 0.04
Generate the monthly payroll checks for an office	1.98	2.00	1.00	1.18	4.00	1.02
Inventory supplies using a hand-held computer	2.57	2.00	2.00	1.21	4.00	0.41
Use a computer program to generate customer bills	2.15	2.00	1.00	1.25	4.00	0.79
Maintain employee records	2.19	2.00	1.00	1.21	4.00	0.71
Compute and record statistical and other numerical data	2.43	2.00	1.00	1.32	4.00	0.51
Operate a calculator	1.96	2.00	1.00	1.14	4.00	1.01
Handle customers' bank transactions	2.10	2.00	1.00	1.25	4.00	0.94
Keep shipping and receiving records	2.30	2.00	1.00	1.18	4.00	0.59
