# Supplementary material for: Evidence for avoidance tendencies linked to anxiety about specific types of thinking
Source: Sci Rep. 2023 Feb 25;13:3294. doi: 10.1038/s41598-023-29834-z (PMC9968294; doi:10.1038/s41598-023-29834-z)
Supplement: Supplementary file 1 — Supplementary Tables. [file 41598_2023_29834_MOESM1_ESM.docx]

**Supplemental Material for “Evidence for Avoidance Tendencies Linked to Anxiety about Specific Types of Thinking”**

Regression model details are shown.

|  | *Regression Models Predicting Affinity Coefficients for Careers* | | | | | | | | | | | | | |
| --- | --- | --- | --- | --- | --- | --- | --- | --- | --- | --- | --- | --- | --- | --- |
| **DV** | a) Creative Affinity Coefficient | | | |  | b) Math Affinity Coefficient | | | |  | c) Spatial Affinity Coefficient | | | |
| **Predictor** | ***b*** | ***se*** | ***t*** | ***p*** |  | ***b*** | ***se*** | ***t*** | ***p*** |  | ***b*** | ***se*** | ***t*** | ***p*** |
| **Creativity Anxiety** | -.237 | .060 | -3.95 | 1E-6 |  | .204 | .061 | 3.34 | .001 |  | .064 | .066 | .98 | .330 |
| **Math Anxiety** | .139 | .054 | 2.58 | .010 |  | -.257 | .055 | -4.68 | 4E-6 |  | .002 | .059 | .03 | .978 |
| **Spatial Anxiety** | -.054 | .060 | -.90 | .367 |  | -.080 | .061 | -1.32 | .189 |  | -.269 | .066 | -4.10 | 5E-5 |
| **General Trait Anxiety** | -.106 | .055 | -1.92 | .055 |  | .085 | .056 | 1.52 | .129 |  | .034 | .060 | .56 | .576 |
| **Non-Creativity Anxiety Control** | .118 | .062 | 1.92 | .056 |  | -.132 | .062 | -2.11 | .036 |  | .122 | .067 | 1.80 | .072 |
| **Gender** | -.370 | .110 | -3.35 | .001 |  | .504 | .112 | 4.50 | 9E-6 |  | -.071 | .121 | -.59 | .556 |
| **Sample** | -.556 | .105 | -5.28 | 2E-7 |  | .119 | .107 | 1.11 | .267 |  | -.137 | .115 | -1.19 | .236 |

**Table S1**

*Note.* Multiple regression models predicting affinity coefficients for careers. All continuous variables are standardized. Gender: 0 is corresponds to female, 1 corresponds to male. Sample: 0 corresponds to the undergraduate sample, 1 corresponds to the MTurk sample. DV = 323 for each model. Model A adjusted *R*^2^ = .196; Model B adjusted *R*^2^ = .171; Model C adjusted *R*^2^ = .036.

|  | *Regression Models Predicting Affinity Coefficients for Activities* | | | | | | | | | | | | | |
| --- | --- | --- | --- | --- | --- | --- | --- | --- | --- | --- | --- | --- | --- | --- |
| **DV** | a) Creative Affinity Coefficient | | | |  | b) Math Affinity Coefficient | | | |  | c) Spatial Affinity Coefficient | | | |
| **Predictor** | ***b*** | ***se*** | ***t*** | ***p*** |  | ***b*** | ***se*** | ***t*** | ***p*** |  | ***b*** | ***se*** | ***t*** | ***p*** |
| **Creativity Anxiety** | -.298 | .058 | -5.13 | 5E-7 |  | .303 | .060 | 5.07 | 7E-7 |  | -.017 | .063 | -.28 | .783 |
| **Math Anxiety** | .154 | .052 | 2.95 | .003 |  | -.265 | .054 | -4.93 | 1E-6 |  | .062 | .057 | 1.09 | .275 |
| **Spatial Anxiety** | -.015 | .058 | -.25 | .803 |  | -.070 | .060 | -1.18 | .238 |  | -.184 | .063 | -2.91 | .004 |
| **General Trait Anxiety** | -.114 | .053 | -2.13 | .034 |  | .132 | .055 | 2.41 | .017 |  | -.123 | .058 | -2.11 | .036 |
| **Non-Creativity Anxiety Control** | .227 | .060 | 3.80 | 2E-4 |  | -.164 | .061 | -2.68 | .008 |  | .263 | .065 | 4.07 | 6E-5 |
| **Gender** | -.295 | .107 | -2.76 | .006 |  | .256 | .110 | 2.34 | .020 |  | .253 | .116 | 2.17 | .031 |
| **Sample** | -.676 | .102 | -6.62 | 2E-10 |  | .394 | .105 | 3.76 | 2E-4 |  | .091 | .111 | .82 | .411 |

**Table S2**

*Note.* Multiple regression models predicting affinity coefficients for activities. All continuous variables are standardized. Gender: 0 is corresponds to female, 1 corresponds to male. Sample: 0 corresponds to the undergraduate sample, 1 corresponds to the MTurk sample. DV = 323 for each model. Model A adjusted *R*^2^ = .245; Model B adjusted *R*^2^ = .204; Model C adjusted *R*^2^ = .108.

|  | *Logistic Regression Models Predicting Whether Affinity Coefficients for Careers are Negative* | | | | | | | | | | | | | |
| --- | --- | --- | --- | --- | --- | --- | --- | --- | --- | --- | --- | --- | --- | --- |
| **DV** | a) Creative Affinity Coefficient | | | |  | b) Math Affinity Coefficient | | | |  | c) Spatial Affinity Coefficient | | | |
| **Predictor** | ***b*** | ***se*** | ***z*** | ***p*** |  | ***b*** | ***se*** | ***z*** | ***p*** |  | ***b*** | ***se*** | ***z*** | ***p*** |
| **Creativity Anxiety** | .504 | .191 | 2.64 | .008 |  | -.417 | .145 | -2.89 | .004 |  | -.021 | .149 | -.14 | .890 |
| **Math Anxiety** | -.205 | .173 | -1.19 | .236 |  | .431 | .132 | 3.26 | .001 |  | -.040 | .135 | -.30 | .768 |
| **Spatial Anxiety** | .039 | .185 | .21 | .833 |  | .152 | .142 | 1.08 | .282 |  | .340 | .150 | 2.27 | .023 |
| **General Trait Anxiety** | .535 | .170 | 3.15 | .002 |  | -.159 | .132 | -1.21 | .227 |  | -.068 | .139 | -.49 | .624 |
| **Non-Creativity Anxiety Control** | -.454 | .190 | -2.39 | .017 |  | .159 | .145 | 1.10 | .273 |  | -.098 | .149 | -.66 | .512 |
| **Gender** | .832 | .350 | 2.38 | .017 |  | -.731 | .259 | -2.82 | .005 |  | -.170 | .282 | -.60 | .548 |
| **Sample** | 1.548 | .371 | 4.17 | 3E-5 |  | -.120 | .248 | -.48 | .629 |  | .359 | .268 | 1.34 | .180 |

**Table S3**

*Note.* Logistic regression models predicting whether affinity coefficients for careers are negative. Beta estimates are log-odds of having a negative affinity coefficient. All continuous variables are standardized. Gender: 0 is corresponds to female, 1 corresponds to male. Sample: 0 corresponds to the undergraduate sample, 1 corresponds to the MTurk sample. DV = 323 for each model. Model A McFadden’s *R*^2^ = .181; Model B McFadden’s *R*^2^= .077; Model C McFadden’s *R*^2^= .022.

|  | *Logistic Regression Models Predicting Whether Affinity Coefficients for Activities are Negative* | | | | | | | | | | | | | |
| --- | --- | --- | --- | --- | --- | --- | --- | --- | --- | --- | --- | --- | --- | --- |
| **DV** | a) Creative Affinity Coefficient | | | |  | b) Math Affinity Coefficient | | | |  | c) Spatial Affinity Coefficient | | | |
| **Predictor** | ***b*** | ***se*** | ***z*** | ***p*** |  | ***b*** | ***se*** | ***z*** | ***p*** |  | ***b*** | ***se*** | ***z*** | ***p*** |
| **Creativity Anxiety** | .505 | .168 | 3.01 | .003 |  | -.666 | .159 | -4.20 | 3E-5 |  | -.131 | .142 | -.92 | .356 |
| **Math Anxiety** | -.131 | .148 | -.89 | .375 |  | .609 | .140 | 4.35 | 1E-5 |  | -.084 | .130 | -.65 | .519 |
| **Spatial Anxiety** | .048 | .161 | .30 | .764 |  | .212 | .149 | 1.43 | .154 |  | .468 | .147 | 3.18 | .001 |
| **General Trait Anxiety** | .168 | .148 | 1.14 | .256 |  | -.263 | .141 | -1.86 | .063 |  | .351 | .133 | 2.63 | .008 |
| **Non-Creativity Anxiety Control** | -.389 | .166 | -2.34 | .019 |  | .338 | .155 | 2.18 | .029 |  | -.410 | .148 | -2.77 | .006 |
| **Gender** | .755 | .297 | 2.54 | .011 |  | -.402 | .272 | -1.48 | .139 |  | -.343 | .258 | -1.33 | .183 |
| **Sample** | 1.629 | .309 | 5.27 | 1E-7 |  | -.949 | .264 | -3.60 | 3E-4 |  | -.414 | .247 | -1.68 | .093 |

**Table S4**

*Note.* Logistic regression models predicting whether affinity coefficients for activities are negative. Beta estimates are log-odds of having a negative affinity coefficient. All continuous variables are standardized. Gender: 0 is corresponds to female, 1 corresponds to male. Sample: 0 corresponds to the undergraduate sample, 1 corresponds to the MTurk sample. DV = 323 for each model. Model A McFadden’s *R*^2^ = .157; Model B McFadden’s *R*^2^= .143; Model C McFadden’s *R*^2^= .083.

**Table S5**

|  |  |  |  |  |
| --- | --- | --- | --- | --- |
| **DV** | Interest - Careers | | | |
| **Predictor** | ***b*** | ***se*** | ***t*** | ***p*** |
| **Creativity Anxiety * Involves Creative Thinking** | -.009 | .002 | -4.23 | 3E-5 |
| **Math Anxiety * Involves Creative Thinking** | .007 | .005 | 1.24 | .216 |
| **Spatial Anxiety * Involves Creative Thinking** | .001 | .002 | 0.41 | .681 |
| **Creativity Anxiety * Involves Math** | .005 | .002 | 2.65 | .009 |
| **Math Anxiety * Involves Math** | -.025 | .005 | -4.92 | 1E-6 |
| **Spatial Anxiety * Involves Math** | -.005 | .002 | -1.93 | .055 |
| **Creativity Anxiety * Involves Spatial Reasoning** | .005 | .002 | 3.17 | .002 |
| **Math Anxiety * Involves Spatial Reasoning** | .002 | .004 | .53 | .594 |
| **Spatial Anxiety * Involves Spatial Reasoning** | -.006 | .002 | -3.13 | .002 |
| **Involves Creative Thinking** | .367 | .045 | 8.20 | 6E-15 |
| **Involves Math** | .049 | .044 | 1.11 | .267 |
| **Involves Spatial Reasoning** | .029 | .037 | .78 | .436 |
| **Creativity Anxiety** | .001 | .009 | .16 | .874 |
| **Math Anxiety** | .046 | .022 | 2.04 | .042 |
| **Spatial Anxiety** | .033 | .010 | 3.19 | .002 |
| **General Trait Anxiety** | -.008 | .004 | -1.90 | .059 |
| **Sample** | -.611 | .108 | -5.66 | 3E-8 |
| **Gender** | .062 | .073 | .85 | .395 |
| **Age** | -.007 | .005 | -1.53 | .127 |

*Note.* Random intercept, random slope mixed-effects model predicting interest in careers. Variables are unstandardized. Gender: 0 is corresponds to female, 1 corresponds to male. Sample: 0 corresponds to the undergraduate sample, 1 corresponds to the MTurk sample. *N* = 331 participants; 15,880 observations. ICC = .227.

**Table S6**

|  |  |  |  |  |
| --- | --- | --- | --- | --- |
| **DV** | Interest - Activities | | | |
| **Predictor** | ***b*** | ***se*** | ***t*** | ***p*** |
| **Creativity Anxiety * Involves Creative Thinking** | -.012 | .002 | -5.00 | 1E-6 |
| **Math Anxiety * Involves Creative Thinking** | .017 | .006 | 2.84 | .005 |
| **Spatial Anxiety * Involves Creative Thinking** | .002 | .003 | 0.79 | .429 |
| **Creativity Anxiety * Involves Math** | .008 | .002 | 4.21 | 3E-5 |
| **Math Anxiety * Involves Math** | -.024 | .005 | -4.89 | 2E-6 |
| **Spatial Anxiety * Involves Math** | -.002 | .002 | -0.96 | .337 |
| **Creativity Anxiety * Involves Spatial Reasoning** | .001 | .002 | 0.57 | .569 |
| **Math Anxiety * Involves Spatial Reasoning** | .007 | .005 | 1.51 | .133 |
| **Spatial Anxiety * Involves Spatial Reasoning** | -.006 | .002 | -2.73 | .007 |
| **Involves Creative Thinking** | .339 | .052 | 6.57 | 2E-10 |
| **Involves Math** | .013 | .043 | 0.30 | .766 |
| **Involves Spatial Reasoning** | -.036 | .038 | -0.95 | .343 |
| **Creativity Anxiety** | .011 | .009 | 1.17 | .244 |
| **Math Anxiety** | -.013 | .023 | -0.58 | .563 |
| **Spatial Anxiety** | .027 | .011 | 2.55 | .011 |
| **General Trait Anxiety** | -.006 | .003 | -1.66 | .098 |
| **Sample** | -.439 | .088 | -4.96 | 1E-6 |
| **Gender** | .083 | .060 | 1.39 | .165 |
| **Age** | -.007 | .004 | -1.90 | .058 |

*Note.* Random intercept, random slope mixed-effects model predicting interest in activities. Variables are unstandardized. Gender: 0 is corresponds to female, 1 corresponds to male. Sample: 0 corresponds to the undergraduate sample, 1 corresponds to the MTurk sample. *N* = 331 participants; 15,887 observations. ICC = .150.
